# Social Media Potential and Impact on Changing Behaviors and Actions in Skin Health Promotion: Systematic Review

**DOI:** 10.2196/54241

**Published:** 2025-01-06

**Authors:** Justyna Martyna Brzozowska, Joanna Gotlib

**Affiliations:** 1 School of Medical & Health Sciences University of Economics and Human Sciences in Warsaw Warsaw Poland; 2 Department of Education and Research in Health Sciences Faculty of Health Sciences Medical University of Warsaw Warsaw Poland

**Keywords:** skin, social media, prevention, behavioral intervention, skin cancer, sun protection, acne

## Abstract

**Background:**

Social media is used as a tool for information exchange, entertainment, education, and intervention. Intervention efforts attempt to engage users in skin health.

**Objective:**

This review aimed to collect and summarize research assessing the impact of social media on skin health promotion activities undertaken by social media users.

**Methods:**

In accordance with the PRISMA (Preferred Reporting Items for Systematic reviews and Meta-Analyses) guidelines, the following scientific databases were searched: Scopus, Web of Science, PubMed, Academic Search Ultimate (via EBSCO), Academic Research Source eJournals (via EBSCO), ERIC (via EBSCO), Health Source: Consumer Edition (via EBSCO), and Health Source: Nursing/Academic Edition (via EBSCO). Using ProQuest Dissertations and Theses, OpenGrey, Grey Literature Report, and MedNar, the search was supplemented with gray literature. Articles on skin care, skin health, skin diseases, skin protection, and educational activities promoting healthy skin on social media were selected for review (search date: February 6, 2023). The following qualification criteria were used: original research; research conducted on social media; and research topics regarding educational activities in skin health promotion, skin care, skin health, skin diseases, and skin protection. To assess the risk of bias, the following tools were used: the Cochrane Collaboration tool for risk-of-bias assessment (randomized controlled trials and quasi-experimental studies) and the Centre for Evidence-Based Medicine checklist (cross-sectional studies).

**Results:**

Altogether, 1558 works were considered, of which 23 (1.48%) qualified, with 3 (13%) studies on acne and 20 (87%) on skin cancer, sunscreen, and tanning. Social media interventions were dealt with in 65% (15/23) of the studies. The review made it possible to investigate cognitive and cognitive-behavioral interventions. In both observational and interventional studies, the most frequently discussed topics were skin exposure and protection against UV radiation and skin cancer. The analyzed research showed that social media is a source of information. Visualization has a strong impact on users. The involvement of social media users is measured through the amount of content shared and contributes to changing attitudes and behaviors regarding skin health.

**Conclusions:**

This review outlined the impact of social media, despite its heterogeneity, on users’ skin health behaviors, attitudes, and actions. It identified strategies for digital interventions to promote skin health. In health sciences, a standardized tool is needed to assess the quality of social media digital interventions. This review has several limitations: only articles written in English were considered; ongoing studies were omitted; and there was a small number of interventional studies on acne and a lack of research on daily skin care, education, and antiaging activities on social media. Another limitation, resulting from the topic being too broad, was a failure to perform quantitative data analysis, resulting in the studies that qualified for the review being heterogeneous.

## Introduction

### Background

For many people, social media platforms are now an integral part of daily life. Social media browsing is the most popular online activity. According to the statistics, >4.26 billion people used social media in 2021, and this number is likely to grow to almost 6 billion in 2027 [1]. In April 2023, a total of 4.8 billion people, or 59.9% of the world’s population, were social media users [2].

Social media is a powerful means of communication, mainly used for an exchange of information and for entertainment. In addition, companies use it for marketing purposes to promote products and services. Currently, there is also a noticeable trend to develop educational platforms, especially on health education. According to Savas et al [3], because of an increase in the number of patients looking for medical information, physicians can use social media such as Facebook as a significant educational tool. Gantenbein et al [4] argue that medical information available on the internet and social media could be useful for most dermatological patients. The authors identified the most important needs of patients: online consultations, medical content on YouTube, and a possibility to chat with a specialist [4]. In turn, according to Banerjee et al [5], social media is a platform for increasing people’s commitment and providing information about UV protection, skin cancer prevention, and its early detection.

The aforementioned research confirms the informational and educational role of social media. The aim of information activities is to increase knowledge about skin cancer and increase awareness of the risks of UV radiation. The effect of these activities should be a change in health behaviors (regular skin photoprotection, regular skin self-examination, and regular monitoring of skin lesions by a physician). Engagement in systematic activities by social media users is crucial.

In addition to its educational role, social media is a tool that supports life and work. Rew et al [6] created a prototype of an application based on content concerning social media users’ daily activities. The application can assess the condition of the skin and provides advice on improving it, with suggestions for care products.

In addition, social media can be a motivational tool by motivating systematic care and protection activities aimed at healthy and well-groomed skin.

Focusing mainly on the analysis of social media content, previous reviews have discussed their impact on skin care [7] and skin cancer prevention [8]. They have also discussed the effects of social networking sites on health behavior change [9] and on available digital interventions, mainly stressing the importance of sun protection and skin self-examinations [10].

Unlike previous systematic reviews, we sought to understand the nature of motivation to act. We were interested in the following aspects: (1) What dermatological problem that is talked about on social media is the main area of interest for researchers? (2) What drives social media audiences to take goal-directed actions? (3) What is the level of involvement of social media users in achieving this goal? (4) What contributes to perseverance in pursuing a goal? The goal is healthy and well-groomed skin. To achieve this goal, health behavior changes must be made.

We wanted to draw attention to the role of social media as a tool for collecting data and a tool that encourages users to take actions to care for their skin (including preventing the development of skin cancer and alleviating the symptoms of dermatological diseases).

The main parts of skin health preventive measures are promoting skin care, skin protection, and control routines. The involvement and motivation of social media users is important. Therefore, research is needed to conduct a systematic review that evaluates the impact and effectiveness of social media in this field. In addition, such research should not only focus on the information provided by social media but also try to engage users in actions aimed at skin health.

This review helps understand and explores what is already known about social media potential and its impact on users’ skin care behaviors and skin health preventive measures. This study suggests the need to optimize social media interventions and develop new studies that will assess long-term change in skin health behaviors. In addition, by increasing the importance of skin protection and its diseases, this review has practical significance. The results of this research can be helpful in practice and in research investigating the role of social media in preventive health care. This review may support the work of dermatologists, cosmetologists, and health educators because it provides information they need, sometimes on complex topics, in a simple and easily available way.

The novelty of this review is that, through the heterogeneity of the research, a broader view of the impact of social media on skin health behaviors is presented. This study provides new observations about the prespecified desired outcome to be achieved. It complements the theory of engagement with digital interventions.

This study differs from previous ones in that it presents social media as an important digital stimulus. It affirms the integration of motivation into specific physical, affective, and cognitive action. The empirical observations concerning, on the one hand, the demonization of the effects of the sun on the skin and, on the other, the image of an attractive tan, which occur in a cause-effect conflict, are surprising.

This review proves that the image, not just the content, is of great importance. There is a noticeable trend in which the image becomes more important than scientific reports. This review also presents the negative side of social media.

### Objectives

This paper presents a systematic review of the literature investigating the role of social media platforms as an educational and intervention tool in promoting preventive skin health measures. The aim of this review was to collect and summarize research assessing the impact of social media on skin health promotion activities undertaken by social media users. In the analyzed studies, we focused on the reception of information about the skin (eg, skin cancer, acne, and tanning) by social media users. The literature review comprised (1) studies assessing the impact of the content of social media on its users, (2) social media intervention studies, and (3) studies determining the impact of posts generated by users on their health decisions and their self-monitoring.

The following research questions were formulated:

1. What are the existing data on the use of social media as an educational intervention tool in skin health preventive measures?
2. What activities on social media can be effective in promoting skin health?
3. What intervention strategies are disseminated via social media and what is their role in promoting skin health?

## Methods

### Design

This systematic review was conducted in accordance with the PRISMA (Preferred Reporting Items for Systematic reviews and Meta-Analyses) guidelines [11,12] and with the Synthesis Without Meta-Analysis reporting guideline [13]. The PRISMA guidelines ensure the highest quality in scientific research. To more fully present the results of the review, a narrative form was additionally adopted.

### Information Sources and Search Strategies

This review was conducted using the following databases: Scopus, Web of Science, PubMed, Academic Search Ultimate (via EBSCO), Academic Research Source eJournals (via EBSCO), ERIC (via EBSCO), Health Source: Consumer Edition (via EBSCO), and Health Source: Nursing/Academic Edition (via EBSCO). In addition, the search was supplemented with gray literature using ProQuest Dissertations and Theses, OpenGrey, Grey Literature Report, and MedNar. A reference list search for relevant articles was also conducted.

The search strategy was first constructed by the authors and then consulted with a librarian experienced in scientific information extraction. The developed phrase was as follows:

((“social media” OR “Facebook” OR “Instagram” OR “Twitter” OR “Pinterest” OR “YouTube” OR “TikTok” OR “SnapChat”) AND (“skincare” OR “skin care” OR “skin health” OR “skin diagnosis” OR “skin type” OR “beautician” OR “cosmetologist” OR “cosmetician” OR “cosmetic consultation*” OR “cosmetic dermatology” OR “cosmetic product*” OR “dermocosmetic*” OR “cosmetic procedure*” OR “skin cleans*” OR “skin moistur*” OR “skin massage” OR “facial cleans*” OR “facial moistur*” OR “facial massage” OR “photoprotection” OR “sunscreen*” OR “sun protection” OR “skin hydration” OR “skin rejuvenation” OR “skin cancer” OR “skin prevention” OR “acne” OR “rosacea” OR “anti-acne” OR “anti-aging” OR “anti-wrinkle” OR “skin aging” OR “oily skin” OR “dry skin” OR “combination skin” OR “sensitive skin” OR “pigmented skin” OR “pimple*” OR “pustule*” OR “papules” OR “blackheads”))

The search was conducted on February 6, 2023, and on the same day, the records were imported from the databases into the Mendeley reference manager (Elsevier). The search was not limited to a period to obtain as many results as possible.

### Study Selection

After applying the search strategy, the studies were imported and saved to the Mendeley reference manager, and then duplicates were removed. The selection of the studies consisted of 2 stages. The first stage was conducted by one of the authors (JMB), who considered the title and abstract to identify and exclude irrelevant studies. To reach a mutual agreement, the other author (JG) reviewed a randomly selected sample containing 10% of the titles and abstracts. When, based on the title and abstract, the decision whether to include a work was difficult, the full article was then downloaded. The second stage of the selection consisted of an analysis of the full texts of the articles by each author (JMB and JG) separately to determine whether they could be included, and the reasons for exclusion were listed. When the decision to include or exclude a study was unclear, the authors clarified ambiguities and different opinions through discussions. This stage of the article selection process required both authors to be present to resolve voting conflicts. All studies were assessed using the inclusion and exclusion criteria described in the next section.

### Inclusion and Exclusion Criteria

All the inclusion and exclusion criteria were defined a priori (Textbox 1).

In particular, the following papers were excluded during the first stage of the selection process: papers written in a language other than English, papers not related to topics of skin care and skin health promotion on social media, conference papers, information from beauty and health blogs, posters of prevention campaigns, and interviews and articles from popular science magazines advertising a specific cosmetic brand. In addition, articles on social media safety rules, product protection, ethics, diet, COVID-19, and tele-dermatology and materials related to patient consent to post photos on social media were eliminated. During the second stage, articles were allocated to the following groups: articles fully meeting the inclusion criteria, comments, case studies, review papers, research protocols, papers in which skin care and skin health preventive measures were not the main topic, papers in which social media was not the main topic (eg, it was used to recruit study participants or to disseminate survey results), papers on plastic surgery and body image perception on social media, papers on social media marketing (eg, dealing with the impact of advertisements encouraging consumers to purchase cosmetic products or with methods of promoting the sale of cosmetic products or assessing the satisfaction with cosmetic products and with brand image building of skin care products), papers on the psychological effects of social media (eg, self-acceptance of social media users; self-confidence; and the relationship among emotions, emotional states, and the use of social media), papers on social media search for medical information by dermatology patients, and papers focusing exclusively on the analysis of the content most frequently shared on social media (ie, posts and videos).

Articles with educational value and assessing the impact of social media skin-related information on its users were included. This review also included studies that investigated social media interventions (articles on behavioral interventions aimed at skin health promotion, which were included during the primary analysis) and studies that investigated the impact of social media data on users (articles on skin health promotion, which were included during the secondary analysis).

**Inclusion and exclusion criteria.**

**Inclusion criteria**

- Papers based on original research (ie, research articles)
- Papers on skin care, skin health, skin diseases, and skin protection and papers on educational activities directed at skin health preventive measures
- Papers on social media (eg, Facebook, Instagram, Twitter [subsequently rebranded X], Pinterest, YouTube, TikTok, and Snapchat)

**Exclusion criteria**

- Papers written in a language other than English
- Review papers
- Comment papers
- Case studies
- Conference papers
- Clinical trial reports
- Information from beauty and health blogs, popular science magazines, interviews, and posters from prevention campaigns
- Papers in which social media coverage of skin care, skin health, and preventive actions were not the main topic

### Data Extraction

Research information from the included articles downloaded during the search process was rechecked by both authors (JMB and JG) to ensure that it met the inclusion criteria. The following details were extracted from each paper: authors, year of publication, geographic location of data collection (country where the research took place), type of social media platform, form of information, number and characteristics of study participants, type of study or data collection methods, and main topic and purpose of the research (Multimedia Appendix 1 [14-36]). In addition, one author (JMB) developed a form with questions (Textbox 2) in which both authors independently recorded their responses (summarized in Multimedia Appendix 2 [14-36]). The PRISMA checklist is provided in Multimedia Appendix 3.

**Questions related to qualified publications.**

What are the authors and the year of publication?

- Is intervention a topic of the study?
- Does the study evaluate the impact of social media on users?
- Does the study deal with public health campaigns?
- Does the study mention skin self-examination?
- Does the study mention the use of cosmetic materials (eg, sunscreen)?
- Does the study contain a statistical analysis?
- What are the main results of the study?
- What are the conclusions or opinions of the authors on the effectiveness of information delivery strategies on social media?

### Quality Assessment

The methodological quality of each study included in this review was assessed separately by both authors (JMB and JG), and their opinions were compared. In case of disagreement, the authors discussed their doubts and came to a consensus. The Cochrane Collaboration tool for risk-of-bias assessment was used to assess the risk of bias in randomized controlled trials and quasi-experimental studies [37]. A total of 6 domains were evaluated, with the plus sign (+) representing a low risk of bias, the en dash (–) representing a high risk of bias, and both (+ and –) representing an unclear risk of bias. Blinding of participants and staff was not assessed as this was considered impossible in some studies. A study that received a score of ≥4 plus signs was considered to be of high quality.

For cross-sectional studies, the Centre for Evidence-Based Medicine checklist for critical appraisal was used [38]. It consists of 12 questions with possible answers of “yes,” “cannot tell,” or “no.” A cutoff value of 75% was used for the assessment. This means that, with ≥9 affirmative responses, the study was considered to be of high quality. Otherwise, the study was considered to be of low quality.

### Data Synthesis Strategy

A narrative synthesis of the included studies was performed in accordance with the recommendations by Popay et al [39]. During data analysis, the authors considered the possibility of classifying the included publications according to the health problem. However, after reanalysis of the selected publications, the authors decided that a better conceptual model was to group them according to the type of intervention, if it was used.

## Results

### Search Results

**Figure 1:**
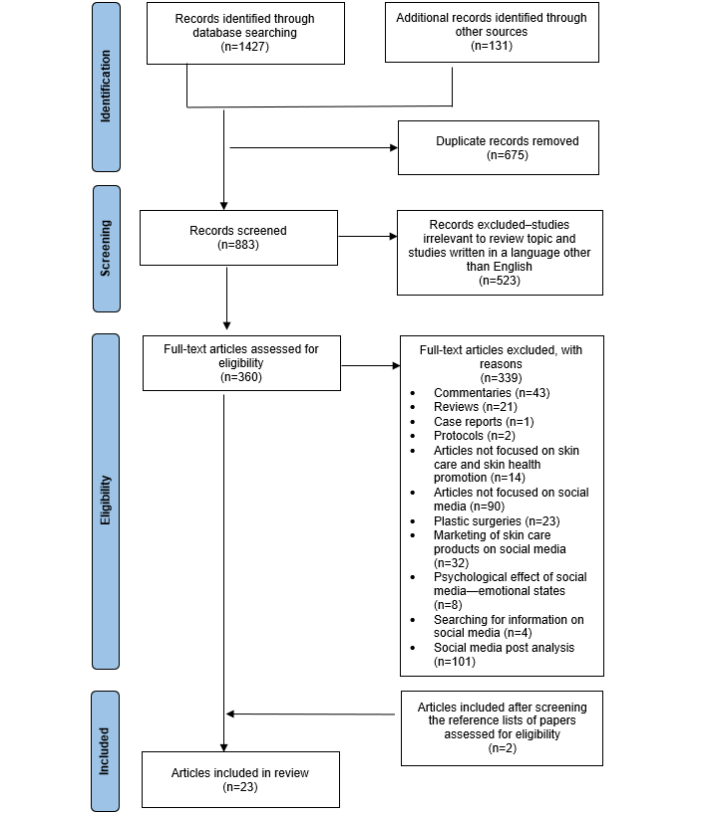
PRISMA (Preferred Reporting Items for Systematic reviews and Meta-Analyses) flow diagram.

A literature search revealed 1558 records (Scopus: n=485, 31.13%; Web of Science: n=305, 19.58%; PubMed: n=270, 17.33%; Academic Search Ultimate via EBSCO: n=234, 15.02%; Academic Research Source eJournals via EBSCO: n=26, 1.67%; ERIC via EBSCO: n=10, 0.64%; Health Source: Consumer Edition via EBSCO: n=35, 2.25%; Health Source: Nursing/Academic Edition via EBSCO: n=62, 3.98%; ProQuest Dissertations and Theses: n=22, 1.41%; OpenGrey: n=4, 0.26%; Grey Literature Report: n=0; MedNar: n=105, 6.74%). During the identification stage, 91.59% (1427/1558) of the records were obtained from scientific databases, and 8.41% (131/1558) of the records were obtained from gray literature. After removing 43.32% (675/1558) of duplicates, 883 records remained. After reviewing the titles and abstracts of these 883 records, 523 (59.2%) were excluded because they were irrelevant to the review topic or were written in a language other than English. A total of 40.8% (360/883) of the publications were selected for full-text download. After analyzing the full texts of the articles, 339 were excluded (n=43, 12.7% comments; n=21, 6.2% reviews; n=1, 0.3% case reports; n=2, 0.6% protocols; n=14, 4.1% articles not focused on skin care and skin health promotion; n=90, 26.5% articles not focused on social media; n=23, 6.8% articles about plastic surgeries; n=32, 9.4% articles about marketing of skin care products on social media; n=8, 2.4% articles about the psychological effect of social media—emotional states; n=4, 1.2% articles about searching for information on social media; and n=101, 29.8% articles about social media post analysis). These studies did not meet the inclusion criteria. A total of 21 articles met the inclusion criteria. In addition, after a detailed analysis of the eligible studies and their references, 2 publications were added. In total, 23 publications were included in this review (Figure 1).

### Study Characteristics

The detailed characteristics and main results of the studies included in this systematic review are presented in Multimedia Appendix 1 [14-36] and Multimedia Appendix 2 [14-36]. Most of the research was conducted using Facebook. Of the 23 studies, most (n=14, 61%) were conducted in the United States [14-27], with a smaller number conducted in other countries: the United Kingdom (n=3, 13%) [28-30], Australia (n=3, 13%) [31-33], the Netherlands (n=1, 4%) [34], Denmark (n=1, 4%) [35], and Saudi Arabia (n=1, 4%) [36]. The highest number of studies was published in 2022 (5/23, 22%) [16,17,21,22,36], 17% (4/23) of the studies were published in 2020 [19,27,28,30], 13% (3/23) were published in 2019 [20,26,32], 13% (3/23) were published in 2018 [14,18,24], 13% (3/23) were published in 2017 [29,31,34], 9% (2/23) were published in 2021 [15,25], 9% (2/23) were published in 2011 [33,35], and 4% (1/23) were published in 2016 [23]. The oldest included publications dated back to 2011 (2/23, 9%) [33,35].

The vast majority of the studies were on skin cancer, sunscreen, and tanning (20/23, 87%), with only 13% (3/23) of the studies on acne [19,27,36]. A total of 17% (4/23) of the studies dealt with skin self-examination [18,19,30,34]. On the other hand, 43% (10/23) of the studies mentioned the use of cosmetic products [14,18,21,22,25,27,28,31,33,36], whereas 35% (8/23) of the studies recommended sunscreen use [14,18,21,22,25,28,31,33]. In addition, 43% (10/23) of the studies discussed the impact of public health campaigns [15-17,20,22,28-30,33,35]. It is worth noting that several publications discussed the results of the same research (eg, 3/23, 13% of the studies by Buller et al [15-17])

Among the 23 studies included in this review, social media interventions were the topic of 15 (65%) [15-18,20-22,24,25,28,29,32-35], among which cognitive effects were assessed by Agha-Mir-Salim et al [28], Damude et al [34], Gough et al [29], and Mingoia et al [32]. On the other hand, both cognitive and behavioral effects were assessed in the studies by Buller et al [15-17], Coups et al [18], Køster et al [35], Morrison et al [20], Myrick et al [21], Pagoto et al [22], Potente et al [33], Stapleton et al [24], and Vraga et al [25]. Comparison groups were used by Agha-Mir-Salim et al [28], Buller et al [15-17], Gough et al [29], Køster et al [35], Mingoia et al [32], Morrison et al [20], Myrick et al [21], Pagoto et al [22], Potente et al [33], and Vraga et al [25].

### Quality Appraisal

The risk-of-bias assessment for randomized controlled trials is summarized in Table 1. A total of 35% (8/23) of the studies turned out to be of high quality [15-17,20,22,25,28,32], and 4% (1/23) of the studies were of low quality [29]. A total of 57% (13/23) of cross-sectional studies were of high quality [14,18,19,21,23,24,26,27,30,31,33,35,36], and 4% (1/23) of cross-sectional studies were of low quality [34]. A summary is provided in Multimedia Appendix 2 [14-36].

**Table 1:** Cochrane risk-of-bias assessment.

Study	Random sequence generation	Allocation concealment	Blinding of participants and personnel or blinding of outcome assessment	Incomplete outcome data	Selecting reporting	Other bias
Agha-Mir-Salim et al [28], 2020	–^a^	+^b^	–	+	+	+
Buller et al [15], 2021	+	+	+	+	+	– (publishing the results of the same study multiple times)
Buller et al [17], 2022	+	+	+	+	+	– (publishing the results of the same study multiple times)
Buller et al [16], 2022	+	+	+	+	+	– (publishing the results of the same study multiple times)
Gough et al [29], 2017	+	–	–	+	+	– (IP addresses were not checked for duplicate users)
Mingoia et al [32], 2019	+	+	–	+	+	+
Morrison et al [20], 2019	+	+	+	+	+	+
Pagoto et al [22], 2022	+	+	+	+	+	+
Vraga et al [25], 2022	+	+	+	+	+	+

^a^High risk of bias.

^b^Low risk of bias.

### Data Synthesis

#### Overview

Among the assumptions we made for this review was that skin health promotion on social media is a broad concept that includes regular activities aimed at protecting the skin against the risk of developing skin cancer, as well as promoting skin care that can ensure good skin condition, prevent skin diseases, slow down the skin aging process, and keep the skin looking nice, which can contribute to one’s well-being. However, most of the studies included in this review (20/23, 87%) focused on behaviors associated with skin cancer risk and prevention (exposure to and skin protection from UV radiation). Skin cancer was the main area of interest for researchers, undoubtedly because it is a high-profile problem. There were not enough studies on other skin conditions to draw firm conclusions about the effects of social media. Nevertheless, it is possible to analyze and compare the effect of information and motivational activities on social media on the engagement of users and their behavior changes and actions taken. This will enable social media moderators to develop overall motivational intervention strategies.

This review included observational studies in which social media was used as a tool to disseminate information about the skin, as well as intervention studies in which social media content was manipulated. Despite the fundamental differences in the methodology of these 2 types of research, the main goal was the same—skin health promotion. Due to differences in research methodology, comparison and summary were difficult.

The results are presented in the following sections in narrative form. A division was made into studies that did not use an intervention and studies that did use an intervention. Then, to synthesize and integrate the results well and draw consistent conclusions, observational studies and then interventional studies were summarized and compared. Attention was drawn to the limitations of observational studies compared to interventional studies.

#### Assessing the Impact of Social Media on Skin Care and Skin Health Promotion Without an Intervention

The studies included in this review argued that social media is a useful source of knowledge about skin health. According to Bahaj et al [36], most individuals aged between 18 and 25 years choose social media as their first source of acne treatment advice. However, much of social media advice does not follow the current guidelines of the American Academy of Dermatology [40]. The aforementioned authors also found that 74.1% of those social media users had a college degree. There was a positive link between educational attainment and willingness to use social media for advice (*P*=.002). In addition, a significant link between the severity of acne and search for treatment advice on social media was found (*P*<.001) [36].

Similarly, in the study by Yousaf et al [27] with 130 participants with acne, 45% (n=58) of them used social media for advice on its treatment. Of these 58 participants, 72% were women (n=42), who were 75% more likely to use social media for advice on acne treatment (prevalence ratio=1.75, 95% CI 1.11-2.76; *P*=.01). In addition, only 31% of the respondents who consulted their problem on social media managed their condition in line with the American Academy of Dermatology clinical guidelines [27]. However, both Bahaj et al [36] and Yousaf et al [27] observed that the advice available on social media did not comply with the guidelines of the American Academy of Dermatology [40]. In the study by Basch et al [14] on skin cancer and tanning, it was found that people who did not fully adhere to proper sunscreen behavior were more likely than those who followed sun protection rules to believe that social media was an accurate source of health information (10.5% vs 3.3%; *P*=.046) or a helpful source of health information (62.5% vs 46.7%; *P*=.02).

According to Mingoia et al [31], the level of social media use was significantly and positively correlated with users’ sun exposure (*P*<.001), sunburn (*P*<.001), and dissatisfaction with skin tone and negatively correlated with sun protection (*P*<.001). This meant that viewing photos, posting photos, sharing content, and the number of likes were significantly linked with greater sun exposure, lower sun protection, and greater dissatisfaction with skin tone. It is worth mentioning that the aforementioned study drew attention to the fact that women, to a greater extent than men, are image oriented and more attracted to visualization on social media. For them, the use of images in health messages may be effective in preventive skin health care campaigns [31].

In the study by Stapleton et al [23], 45.8% of participants had a college degree, and 14.8% had a bachelor’s degree or higher (a sample of 463 participants). The authors found that higher rates of indoor tanning were associated with Twitter (subsequently rebranded X) and Instagram use among a sample of young adult women. As a result, the authors argued that the aforementioned social media platforms could be a valuable way to provide information about skin cancer prevention among compulsive indoor tanners [23].

In contrast, the sample in the study by Willoughby and Myrick [26] consisted of 502 female college students aged between 18 and 29 years, and it was found that participants who reported more frequent use of social media were more likely to sunbathe outside. Thus, the researchers found that greater use of visual social media platforms such as Instagram, Snapchat, and Pinterest was likely to increase tanning [26].

According to the studies by Bahaj et al [36], Yousaf et al [27], Basch et al [14], Mingoia et al [31], Stapleton et al [23], and Willoughby and Myrick [26], widely shared social media content could influence engagement in risky health behaviors, affecting users’ knowledge, attitudes, and actions. Despite the negative impact of social media, the authors noted that it could be a powerful informational, educational, and intervention tool for preventive skin health care campaigns. In addition, the identification of the target group of health advice recipients made it possible to fight disinformation using specific prevention channels (ie, social media platforms). In contrast to the aforementioned studies, the studies by Guckian et al [30] and Martel et al [19] mainly pointed to the positive impact of social media.

Guckian et al [30] found that interventions via social media could have a positive impact on health-related behaviors. According to the authors, social media could motivate patients with distressing cutaneous lesions to visit melanoma screening clinics. However, the authors found that the most common motivating reason for visiting a melanoma screening clinic was skin self-examination. In this study, 65% (162/249) of the patients were regular users of social media, 33% (83/249) saw posts on the internet about cutaneous lesions, 10% (8/83) saw posts from physicians, 36% (30/83) saw posts from health authorities, and 37% (31/83) saw posts from other people (social media users). In addition, 33% (83/249) of patients searched for information about their cutaneous lesions on the internet, whereas one patient did so on social media. In addition, 24% (6/25) of patients said that more posts on cutaneous lesions should be shared [30].

Martel et al [19] found that social media use could be a motivating factor. According to the authors, editing one’s own cutaneous lesions on social media, in particular acne and acne scars, contributed to an increase in users’ awareness and encouraged them to seek dermatological care. Of 145 people who edited their skin lesions, as many as 128 (88.3%) edited acne and acne scars. This editing increased their awareness of the need for dermatological care (*P*=.02) [19].

#### Assessing the Impact of Social Media on Skin Care and Skin Health Promotion After an Intervention

##### Comparative Studies on the Role of Digital Social Media Interventions and Traditional Media in Health Promotion

The study by Agha-Mir-Salim et al [28] was one of those comparing the effects of digital interventions with those of printed materials. The research involved the SunSafe campaign that aimed to raise awareness of sun exposure risk, melanoma, and the need for sun protection among individuals aged 18 to 29 years. Using flyers and Facebook, the authors found that, after the intervention, the average knowledge score improved in both groups (Facebook=1.82; flyers=3.04; *P*<.001). However, printed flyers turned out to be more effective than Facebook posts (95% CI 0.35-2.09; *P*=.006) [28].

##### Social Media Interventions as an Educational Strategy

Damude et al [34] assessed the knowledge of patients with melanoma about their illness and their opinion on multiple ways of providing information. YouTube instructional videos presented in the research were designed to stress the need for self-examination of the skin and lymph nodes and demonstrate how to do this properly. It turned out that 63% of patients preferred to receive information in multiple ways, 92% of them preferred verbal instructions from their physician, 62% preferred educational YouTube videos, and 43% preferred to receive instructions via brochures. In addition, the authors concluded that there was an urgent need for educational activities focused on melanoma and for supplementing the methods of education with instructional YouTube videos [34].

In the study on skin cancer prevention by Gough et al [29], a trend toward increasing knowledge about preventive actions in response to social media educational interventions was observed. Those interventions increased awareness that fair-skinned people required the most protection and that skin cancer was the most common form of cancer, with melanoma being its most serious type. In addition, the authors noted a trend of increasing awareness of sun and UV ray exposure, skin cancer risk, and the need for sun protection.

Using an educational strategy and cognitive dissonance, Mingoia et al [32] investigated the effects of a social media intervention on the opinion that *tanned skin is desirable and beneficial for appearance*. The educational strategy was aimed at reducing positive attitudes toward sunbathing and improving the ability to critically analyze social media content [32].

The study by Pagoto et al [22] is another example of a dissonance-based social media intervention to promote sun safety. The intervention involved encouraging survey respondents to create social media posts on healthy skin or a healthy lifestyle. According to the authors, participants in the healthy skin intervention group reported a higher motivation to use protective clothing and sunscreen and a decreased motivation to tan. The authors suggested that this intervention, encouraging social media content creation and response, should enhance cognitive dissonance and affect social norms, which in turn might change attitudes and behaviors concerning tanning [22].

The study by Stapleton et al [24] is another example of a dissonance-based intervention focusing on healthy body image. As part of this intervention, a group of Facebook users was encouraged to publish posts and share their opinions on them. The aim of the dissonance-inducing intervention approach was to engage participants in group discussions related to body image [24].

The topic of skin cancer prevention and the use of tanning beds was also addressed by Morrison et al [20]. To educate the audience, the authors used 3 different short videos aimed at discouraging indoor tanning. The first was a humorous video created in collaboration with a physician, the second was a music video with an Instagram star, and the third one was based on facts about skin cancer. The humorous video received the highest engagement, measured via the number of comments, reactions, and shares, whereas the fact-based video received the lowest engagement [20].

Moreover, in the study by Potente et al [33], an ironic music video was also used, the aim of which was to engage young people in skin cancer prevention. Different forms of entertainment, education, and marketing were used on social media to change attitudes and behaviors regarding sun protection [33].

In contrast to the aforementioned studies, Vraga et al [25] used a video with incorrect information about sunscreen and skin cancer. The results indicated that the disinformation video, compared to an educational video, increased belief in sunscreen myths (*P*<.001) and decreased intent to use sunscreen (*P*<.01) [25].

In the study by Myrick et al [21], an intervention focusing on appearance and self-control–related emotions was used. The results indicated that the intervention focused on appearance contributed to a reduction in the time spent viewing Instagram images of tanned women. In contrast, after the intervention involving self-control emotions, an increase in anticipated pride and in a positive attitude toward solar safety was observed [21].

##### Digital Interventions on Social Media Based on Social Cognitive Theory, Transportation Theory, and Diffusion of Innovations Theory

Buller et al [15-17] conducted a year-long social media health campaign to reduce mothers’ permissiveness of indoor tanning with their teenage daughters. Mothers became less lenient toward indoor tanning with their teenage daughters immediately after the campaign and 6 months after it ended [15,16]. According to the researchers, social media campaigns could motivate mothers to communicate with their daughters and share posts about the danger of indoor tanning [15]. In turn, Buller et al [17] measured engagement levels by measuring reactions (eg, sadness and number of likes) and mothers’ comments on posts related to the campaign. They noted that users reacted with comments to 76.4% of posts. Immediately after the campaign ended and 32 months after, mothers who posted reactions or comments were less liberal about indoor tanning with their teenage daughters than mothers who did not engage with the posts [17].

##### Social Media Behavioral Interventions

In the study by Coups et al [18], a behavioral intervention was conducted via Facebook targeting young patients with melanoma and their families. The content of the intervention consisted of daily posts about skin cancer risk, total cutaneous exam, skin self-examination, and preventive measures (ie, sun protection). Preliminary analyses by the researchers showed an increase in intentions to use the total cutaneous exam, skin self-examination, and sun protection. In addition, Coups et al [18] found that posts that included personal stories, quizzes, skin care exercises, and an opportunity to ask a question to a specialist were perceived by survey respondents as particularly engaging.

An effective intervention was also demonstrated in the study by Køster et al [35], who reported that, in effect, the use of tanning beds decreased in parallel with antisun campaign activities, with an odds ratio of 0.61 (95% CI 0.54-0.69). The campaign was mainly conducted on social media but also in youth magazines and on the radio. It was supplemented with a music video available on the internet and television on skin damage and the negative effects of using cosmetic beds. In addition, respondents to the postcampaign survey were in favor of legislative changes to restrict the access of children and teenagers aged <18 years to tanning salons [35].

#### Result Summary

To sum up, social media is a place where observational studies and interventional studies can be carried out. Unlike interventional studies, observational studies do not assess cause and effect relationships, require a large sample, and carry a high risk of error. In both observational and interventional studies, the most frequently discussed topics were skin exposure to and protection against UV radiation and skin cancer. Only 13% (3/23) of observational studies focused on acne [19,27,36]. No skin care studies were found that met the inclusion criteria. Observational studies conducted on social media showed that social media is a source of information [14,23,26,27,30,36] and a place where one can edit one’s own photos [19,31]. Observational studies on acne showed that dissatisfaction with the appearance of the skin (presence of skin lesions) stimulates individuals to use social media. In the case of observational studies on exposure and protection against UV radiation, it was shown that the use of social media increases dissatisfaction with appearance and, consequently, increases exposure to UV radiation.

**Figure 2:**
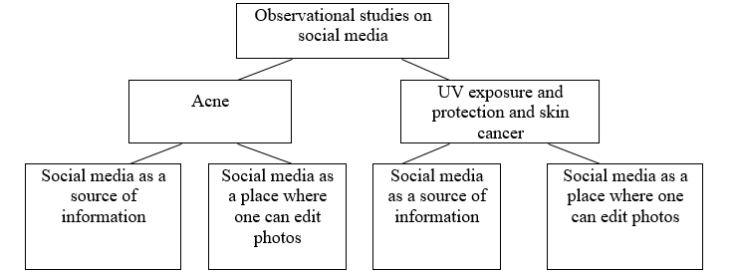
Summary of observational studies on social media.

Visualization in social media has a strong impact on users. Showing images contributes to taking actions that are both beneficial (in the presence of skin lesions)—seeking dermatological care—and unfavorable (the desire to have a nice tan)—increased exposure to UV radiation (Figure 2).

**Figure 3:**
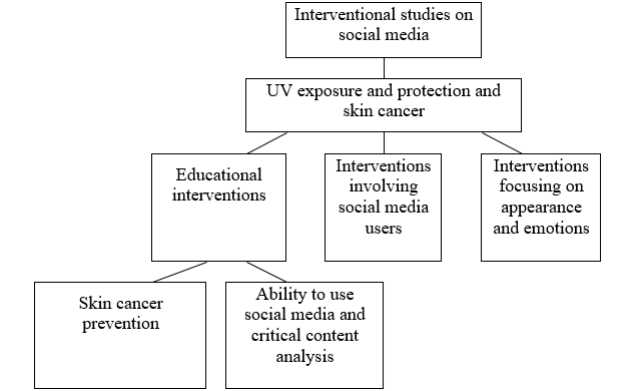
Summary of interventional studies on social media.

The analyzed social media intervention studies mainly concerned skin exposure to and protection against UV radiation, tanning, skin self-examination, and skin cancer. Most studies (11/23, 48%) used educational interventions on skin cancer prevention [15-18,20,25,28,29,33-35]. One study used an educational intervention on social media literacy and critical content analysis [32]. A total of 13% (3/23) of the studies examined social media users’ involvement in generating social media content [15-17,22,24]. These studies pointed out that social media is a motivating factor. Motivation is the readiness to take a specific action, triggered by a need. To take action, one needs the right attitude and the internal conviction that the action is advisable. The aim of educational interventions was to change attitudes toward skin health promotion. However, interventions encouraging participants to create content on social media were intended to engage participants to increase the effectiveness of the intervention and the belief in its purposefulness. A total of 9% (2/23) of the studies focused primarily on body image rather than skin health issues [21,24]. The study by Myrick et al [21] discussed a psychological approach to appearance and emotion self-control (Figure 3). Both in observational and interventional studies, attention was paid to visualization on social media (images, photos, and videos). In addition, attention was paid to the involvement of social media users, which was measured via the amount of content shared, comments, likes, and photos published. Intervention studies showed that educational strategies based on humorous films, interesting personal stories, and opportunities for discussion were more engaging for participants. In observational studies, it was not possible to measure the level of persistence in following recommendations (eg, regular photoprotection). Interventional studies provided this possibility, and their results can be checked after a period.

## Discussion

### Principal Findings

The results of this review indicate that social media is a platform for disseminating health messages on an ongoing basis and for creating interventions targeted at specific groups. However, research on social media impact on education, attitudes, behaviors, and actions concerning skin health promotion was inconclusive and varied. Some studies (2/23, 9%) stressed the negative side of social media, noting that, for example, its acne advice was inconsistent with current guidelines [27,36]. Others (4/23, 17%) drew attention to the fact that social media can promote risky health behaviors concerning sunbathing without sunscreen, which may result in skin cancer [14,23,26,31]. On the other hand, without focusing on digital interventions, some of the studies included in this review (2/23, 9%) dealt with a positive impact of social media on skin care, skin protection, and skin health promotion. They noted that social media increases users’ self-awareness and motivates them to take care of their skin [19,30]. According to Benetoli et al [41], social media has positively affected users’ interaction with health care professionals. As a result, patients feel more confident and more assertive in their decision-making [41]. Most of the studies discussed in this review (14/23, 61%) argued that social media interventions resulted in positive effects on preventive skin health measures, changing users’ behaviors, actions, attitudes, and knowledge levels [15-18,20-22,24,28,29,32-35]. Therefore, it is reasonable to ask how to make social media interventions effective and how to achieve a long-term change in user behaviors.

Agha-Mir-Salim et al [28] investigated the effects of social media digital interventions and found that Facebook was less effective than printed materials in improving awareness of skin cancer and the need for sun protection. However, without information about the level of audience engagement measured through the number of posts and reactions to them, it is difficult to explain the reasons why flyers were more effective than Facebook [28].

User engagement is critical to the success of social media interventions. The assessment of user engagement with social media interventions was presented in 35% (8/23) of the studies, determining the reactions to posts using the number of likes, comments under posts, and shares [17,18,20,22,24,29,32,33]. In addition, the concept of social media engagement plays a key role in health psychology [42]. Systematic reviews evaluating strategies that promote technology-based engagement have shown a potential positive impact of these strategies on health behaviors [43-47]. The success of social media engagement is based on its effect on society. Psychological factors and neural mechanisms are important [48]. Nahum-Shani et al [48] explain how positive engagement develops in response to a digital stimulus facilitating behavior change. By describing the neural basis of decision-making (by explaining its application to digital interventions), the researchers extend the concepts by Samanez-Larkin and Knutson [49] (affect-integration-motivation), creating the concepts of affect-integration-motivation and attention-context-translation of motivation to behavior. These additional elements can be used for targeted engagement of social media users with digital interventions [48].

Behavioral interventions through social media have many advantages, such as a large audience, low costs of disseminating information, and a possibility to create individual content and interact with other users with similar experiences or in a similar situation. This allows for mutual support, the exchange of information, and an opportunity to interact with health professionals by consulting specialists and obtaining answers [18].

In the observational studies on acne by Bahaj et al [36] and Yousaf et al [27], the authors pointed out that much of the advice available on social media is not consistent with the guidelines of the American Academy of Dermatology. In addition, in the observational study on skin cancer by Basch et al [14], it was shown that people who believed that social media was an accurate source of health information were more likely to fail to follow proper sun protection practices. The aforementioned study showed that social media can increase belief in health myths. The study by Vraga et al [25] showed that videos with erroneous information had a stronger impact on users than educational videos. Social media actions should also be aimed at combating misinformation. The content posted should be in agreement with evidence-based medicine and with the guidelines of the American Academy of Dermatology. In addition, interventions should increase the knowledge of social media users about the dangers of improper skin care and change their positive attitude toward inappropriate activities such as too much tanning. Social media interventions should also contribute to an increase in assertiveness toward inappropriate, harmful content, building self-esteem in users and teaching critical analysis of the content shared. In the case of tanning, interventions should describe alternative options, such as the use of safe moisturizing bronzers.

It seems that effective interventions should not only aim at the health consequences of poor skin care and poor skin protection but also focus on monitoring skin texture. In addition, they should make an effort to shape social norms regarding attractiveness (ie, by questioning the ideal of tanned skin and promoting sun protection). The use of dissonance induction approaches in interventions also seems to be important. The goal of those approaches is to encourage participants to persuade others to engage in appropriate behaviors [22].

Behavioral health interventions concerning appearance and body image often use persuasive techniques based on cognitive dissonance theories. Those techniques encourage participants to engage in discussions and cognitive exercises. Participants endorse views that contradict their previous beliefs and, in the end, accept these views as their own [24]. According to the dissonance theory, when there is a conflict between a belief (eg, a perfect tan is good) and behaviors (eg, persuading others that a perfect tan must be avoided and sunscreen should be used), psychological discomfort (cognitive dissonance) is the result, which motivates an individual to change their original beliefs (ie, the person is motivated to use sunscreen because tanned skin is less important than healthy skin) [22,50]. The effectiveness of cognitive dissonance interventions has been confirmed in studies on eating disorders [51] and perfect tans [52].

Pagoto et al [53] proposed a methodology for adaptation of social media behavioral interventions. It requires that parameters be defined to determine whether the intervention is based on social media completely or partially. The objective of a social network should be defined, too. First, it should be decided whether the main objective of the intervention is to engage users, disseminate information, or both. The type of intervention should be related to the objective. One-way communication is adopted mainly for public health campaigns. In 2-way communication, the content is generated by both the intervention moderators and the audience. Two-way communication allows for discussions and for help with problem-solving and behavioral advice. When designing interventions, it is important to choose the social networking platform and the target population. Another important element when designing an intervention is content conversion, with short, catchy posts, videos, photos, graphics, and links to articles. When the main focus of the intervention is user engagement, a plan should be constructed. It should include the size of the group, the frequency of posting, and the number of engaged participants, and it should specify whether the posts are automated. In the design of interventions, it is also reasonable to create chat groups or use microcounseling, which involves the intervention moderator initiating the discussion. The engagement plan may also include recruiting friends or family members for the intervention. In addition, it is important to train users on how to clarify expectations and encourage them to express their opinions and publish posts [53].

Almost all the intervention studies analyzed in this review (13/15, 87%) were conducted after Pagoto et al [53] developed their methodology for adapting social media interventions. After analyzing the presented interventions, it can be assumed that the authors followed some of the advice by Pagoto et al [53] when designing their interventions. Intervention studies that focus on engaging participants have found greater intervention effectiveness.

In summary, the analysis of the studies included in this review indicates that, when designing social media interventions for skin health, it is necessary to pretest them in a pilot project with comparison groups, including control groups, and measure user engagement on a regular basis. In addition, designing interventions requires focusing on the content that is designed to engage the audience. Absorbing content should not only be educational but also entertaining and discourage abnormal health behaviors. The content should be focused on the benefits according to positive psychology (to increase its attractiveness); it should focus on thinking about the future using goal setting and motivation (to have nice and healthier skin). It is also reasonable to include personal stories, exercises to engage the audience, and various puzzles. When designing interventions, the theories of social psychology they are based on should be considered.

The results of this review may be useful in creating future skin health promotion programs on social media. It is aimed at all those who are ready to broaden their knowledge and effectively engage in activities related to skin care and skin protection.

This study aimed to fill the research gap in assessing the usefulness of social media for skin health promotion. Its main assumption was that it would contribute to the discussion on the importance of the information provided about the skin and its impact on changing the attitudes and behaviors of recipients of this information.

### Strengths and Limitations

As with any other study, this review had its limitations. The first one was that only articles written in English were analyzed, which meant that important publications written in other languages were not included. The second limitation was the omission of studies that were in progress (not completed). One of them was a Facebook intervention targeted at patients with melanoma and their families (randomized controlled trial) as the report on its results was expected to be completed by November 2023 [54]. Therefore, its analysis was not possible, and this review should be updated at a later time. The third limitation is the small number of interventional studies on acne and a lack of research on rosacea and atopic dermatitis, common conditions dealt with in dermatological and cosmetology clinics. In addition, there was a lack of research on daily skin care, education, and antiaging activities on social media. Some papers, such as those dealing with social media posts on esthetic medicine and more or less invasive plastic surgery, were not included in this review. This prevented a broader analysis and assessment of the impact of social media on users’ skin care and health activities. The last limitation was the failure to undertake a quantitative data analysis because of too broad a topic, which resulted in the heterogeneity of the studies that qualified for analysis.

Despite its limitations, this paper presents an up-to-date review of social media impact on users’ behavior change and the implementation of interventions mainly in the field of skin cancer prevention. In addition, this review identifies research gaps and topics worth focusing on and indicates what should be improved when conducting social media research.

### Conclusions and Practical Implications

Social media remote consultations and digital interventions with the use of new technologies in education and research are a relatively new method of skin health promotion. Despite the limited number of studies and their heterogeneity, this review outlined the impact of social media on user behaviors, attitudes, and actions in terms of skin health. It provided insights into social media importance and identified strategies for digital interventions to promote health. In addition, this review underlines the need to conduct research on skin health promotion via social media and on new methods motivating users to take an active role. There is no doubt that more high-quality studies, consistent and with logical conclusions, on social media using standardized interventions are needed.

The observational studies analyzed in this review (8/23, 35%) showed that social media, according to social media users, is a reliable place to obtain information on skin health. Moreover, there was a noticeable tendency of social media influencing the behavior of social media users. On the basis of unpublished own research (by author JMB), of 150 surveyed social media users (people aged ≤25 years), in 2021, a total of 62% (93 people) believed that information from social media regarding skin cancer prevention, skin care and treatment of acne, and antiaging was very important to them. For 44% of respondents (66 people), information on social media influenced their behavior. In a repeated study in 2023, as many as 86% of respondents (129 people) stated that information on skin cancer prevention, skin care and treatment of acne, and antiaging was very important to them. However, 74% of respondents (111 people) stated that information on social media influenced their behavior.

The studies analyzed in this review showed that more frequent use of social media is significantly associated with more frequent exposure to UV radiation. Social media focuses on visualization. Photos and images are a powerful tool to influence users. In intervention studies, there was a noticeable tendency to encourage people to react to posts and create their own. It can be assumed that interventions involving social media users in generating content increase their motivation to take care of their skin. The aim of social media interventions should be education on promoting skin health based on visualization and motivating social media users to take care of their skin (using photoprotection, limiting sunbathing, using protective clothing, conducting skin self-examinations, and having regular skin checkups with a dermatologist).

The availability of social media worldwide provides unlimited possibilities for transmitting and managing information. Analyzing posts and evaluating user engagement by measuring the number of likes, comments, and followers can be a powerful tool for gathering knowledge and studying patients’ opinions. After becoming familiar with the expectations of social media users and assessing information resources and their quality and credibility, appropriately targeted actions should be taken to educate, encourage, and motivate users to be active in skin care and skin health promotion in everyday life.

Social media has become a tool supporting the life and work of society. The model of Society 1.0 was based on hunting, Society 2.0 was based on agriculture, Society 3.0 was based on industry, and Society 4.0 is based on information. Social media implements the assumptions of the Society 4.0 model through information activities. The vision of Society 5.0 is a balance between technological progress and human needs. Model 5.0 enables the collection of data (eg, on health status) from patients on an ongoing basis. Social media can be a tool for digital interventions and for Society 5.0 implementation. The implementation of Digital Society 5.0 depends not only on the cooperation between countries but also on combined efforts of various branches of medicine, business, and science, which is the basis for creating innovations at both the national and global level. Innovations are not possible without cooperation; the use of global data (big data); and the coordination of many data systems and knowledge platforms, including social media.

Proper and specialized use of social media by dermatologists, pharmacists, cosmetologists, and health educators can increase awareness concerning skin condition and skin health preventive measures. It can encourage users to conduct a skin self-examination and visit a dermatologist. This review could also enable dermatologists, cosmetologists, pharmacists, and health educators to understand social media potential and its impact on users. This review presents social media use for intervention studies and indicates its educational and motivational role in actions related to skin health. In addition, those actions have a clinical significance as social media increases user awareness of skin health promotion. As a result, users are not reluctant to seek medical help. Therefore, this review could be a basis for further research on the role of social media interventions in skin health promotion (including sun protection; mole control; and proper skin care for various dermatological diseases, such as acne, rosacea, or atopic dermatitis). It seems that social media, compared to traditional forms of mass communication, has the potential to make a change in skin care and health behaviors and activities among a larger population.

First, future studies should focus on effective social media strategies to promote skin health. Second, research assessing the effects of health and skin care recommendations would be useful. Third, studies assessing the short-term engagement of social media users would be of practical importance, as well as research assessing their commitment to long-term health and skin care habits. The results of this review, in addition to its theoretical value, could have potential applications in online dermatological and cosmetology consultations and in prohealth campaigns concerning increasingly common skin diseases such as acne and melanoma.

This research dealt with studies investigating individually developed social media strategies. It seems that it would still be appropriate to standardize tools assessing the quality of digital health interventions on social media. Such standardization would be helpful both in the design of new digital interventions and in the evaluation of existing ones.

## Appendix

Multimedia Appendix 1 Characteristics of the included studies.

Multimedia Appendix 2 Findings of the included studies.

Multimedia Appendix 3 PRISMA (Preferred Reporting Items for Systematic reviews and Meta-Analyses) checklist.

## Abbreviations

PRISMA: Preferred Reporting Items for Systematic reviews and Meta-Analyses
