# Supplementary material for: Social Media Potential and Impact on Changing Behaviors and Actions in Skin Health Promotion: Systematic Review
Source: J Med Internet Res. 2025 Jan 6;27:e54241. doi: 10.2196/54241 (PMC11747541; doi:10.2196/54241)
Supplement: Multimedia Appendix 1 [file jmir_v27i1e54241_app1.docx]

Table 2. Characteristics of included studies.

| Author (year) | Country | Platform – Type of social media | Type of data | Patient population (n) | Patient age (years) | Study design; Type of study; Assessment tool | Main topic | Aim(s) |
| --- | --- | --- | --- | --- | --- | --- | --- | --- |
| Agha-Mir-Salim et al. (2020) [28] | United Kingdom | Facebook | Posts;  Printed leaflets | 279 | 18-29-year-old university students or graduates | Randomized controlled trial;  Skin Cancer Questionnaire and Sun Knowledge Questionnaire | Skin cancer/  Melanoma; Sun exposure; Sun knowledge | Evaluating the effectiveness of Facebook, compared to leaflets, in raising awareness of melanoma and harmful sun-related behavior |
| Bahaj et al. (2022) [36] | Saudi Arabia | Instagram  YouTube  Snapchat Twitter  TikTok  WhatsApp  Facebook | Not specified | 4227 participants having experienced acne | At least 13 years old | Cross-sectional study;  Online survey | Acne | Assessing the impact of social media on acne management among the Saudi Arabian population |
| Basch et al. (2018) [14] | United States | Not specified | Posts | 258 | college students | Cross-sectional study;  Survey (questions were adapted from the Health Information National Trends Survey questionnaire and from prior study on sun safety) | Skin cancer;  Sun protection;  Tanning behavior | Exploring factors related to sun protection and tanning behaviors and examining the influence of social media use and information-seeking behaviors on sun protection actions |
| Buller et al. (2021) [15] | United States | Facebook | Posts;  Videos | Mothers (n=869) with daughters aged 14-17 (n=469) | Mothers – not specified; daughters – 14-17 year-old | Randomized controlled trial;  Surveys | Skin cancer;  Indoor tanning | An evaluation of a social media campaign aimed at reducing mothers' leniency towards their teenage daughters' indoor tanning |
| Buller et al. (2022) [16] | United States | Facebook | Posts;  Videos | Mothers (n=869) with daughters aged 14-17 (n=469) | Mothers – not specified; daughters – 14-17 year-old | Randomized controlled trial;  Measurement of reactions (like, sad, etc) and comments | Skin cancer;  Indoor tanning | Assessment of the effects of mothers' involvement on social media campaigns aimed at limiting indoor tanning of their adolescent daughters |
| Buller et al. (2022) [17] | United States | Facebook | Posts;  Videos | Mothers (n=869) with daughters aged 14-17 (n=469) | Mothers – not specified; daughters – 14-17 year-old | Randomized controlled trial;  Surveys | Skin cancer;  Indoor tanning | Examining the long-term effects of a social media campaign for mothers to reduce their adolescent daughters' indoor tanning. |
| Coups et al. (2018) [18] | United States | Facebook | Posts | 48 young-onset melanoma patients and their 40 family members | Not specified | Pilot and Feasibility Study;  Web-based or pencil and paper baseline survey | Skin cancer/Melanoma;  Sun protection;  Total cutaneous exam;  Skin self-exam | Examining the development, feasibility, and preliminary impact of a family-focused Facebook intervention to increase engagement in total cutaneous exam, skin self-exam, and sun protection behaviors |
| Damude et al. (2017) [34] | The Netherlands | YouTube | Videos | 100 melanoma patients | range 20-86 (median age was 60) | 19-item, web-based questionnaire | Melanoma | Investigating knowledge of Dutch melanoma patients and their opinions on the information provision and educational value of YouTube videos |
| Gough et al. (2017) [29] | United Kingdom | Twitter  Thunderclap | Message frames (informative, story, shock, humor);  posts | 337 participants pre campaign;  429 participants post intervention | At least 18 years old | Quasi-experimental feasibility study;  Pre- and post campaign Web-based survey | Skin cancer | Assessment of the feasibility of designing, implementing, and evaluating a social media-enabled intervention for skin cancer prevention |
| Guckian et al. (2020) [30] | United Kingdom | Facebook  Instagram  Twitter  Pinterest | Posts | 249 | Patients of different ages (≥ 18 years old) | Pilot study;  Cross-sectional study;  Quantitative and Qualitative data; Questionnaire | Skin cancer | Identifying the impact of social media on patient presentation to a melanoma screening clinic |
| Køster et al. (2011) [35] | Denmark | MySpace;  Youtube;  Facebook | Posts;  Articles in magazines;  Music video in Internet and television;  Radio | 14514 | 15-59 year-old | Multicenter study;  Cross-sectional study;  Four questionnaires | Skin cancer; Indoor tanning beds | Assessment of the impact of anti sunbed campaign activities on the use of tanning beds |
| Martel et al. (2020) [19] | United States | Instagram | Photograph editing | 257 | Undergraduate students | Survey | Skin lesions;  Acne/acne scars;  Seeking dermatologic care | Evaluating the impact of photo editing on seeking dermatological care |
| Mingoia et al. (2017) [31] | Australia | Not specified | Posts (pictures, text) | 1856 South Australian | Secondary school students (adolescents) | Cross-sectional study;  Survey | Skin cancer;  Sun exposure; Sun protection | Assessing the relationship between social media use and dissatisfaction with skin tone, sun exposure, and sun protection |
| Mingoia et al. (2019) [32] | Australia | Facebook | Posts | 84 women | 18-29 year-old | Pilot study; Randomized Controlled Trial;  Modified version of the Media  Attitudes Questionnaire (MAQ);  7-item tanning attitudes  scale;  Socio-cultural Attitudes Towards Appearance Questionnaire-4 (SATAQ-4);  Physical Appearance Comparisons Scale (PACS);  Modified version of  the Skin Tone Rating Scale | Skin cancer; Tan | Evaluating the effectiveness of a social media literacy intervention delivered via Facebook to reduce positive attitudes towards tanning and improve  ability to approach social media critically |
| Morrison et al. (2019) [20] | United States | Facebook | 3 videos | 7216 | Women aged 18–34 in six states (Alabama, Alaska, Arkansas, Georgia, Kentucky, and Tennessee) | Pilot study;  Randomized controlled trial;  Number of individuals exposed, frequency, duration of views, engagement – number of likes, shares, comments and 3 questions | Skin cancer;  Indoor tanning | Primary aim was to develop engaging skin cancer prevention videos and measure the video reach and engagement;  Secondary aim was to assess the impact of prevention videos on self-reported outcomes related to melanoma risk |
| Myrick et al. (2022) [21] | United States | Instagram | Posts | 120 women | 18-22 year-old | Online questionnaire and Qualtrics questionnaire | Skin cancer; Tan;  Sun-safety attitudes | Assessment of the ability of digital sun-safety interventions to affect self-control-related emotions and visual attention to subsequent tan-ideal images and sun-safety attitudes |
| Pagoto et al. (2022) [22] | United States | Facebook;  Twitter | Posts | 66 tanners | 18-30 year-old | Pilot and Feasibility Study; Randomized controlled trial | Skin cancer;  Healthy Skin;  Healthy Lifestyle | Evaluating the feasibility and acceptability of a dissonance-based social media intervention in which young adults were encouraged to create social media content aimed at promoting sun safety in young adult tanners |
| Potente et al. (2011) [33] | Australia | YouTube  MySpace  Facebook | Video | 1588 (627 respondent s  exposed to the video; 961 respondents not exposed to the video) | 14-24 years | Online surveys and thematic analysis of online conversations;  social media monitoring tools: Radian 6 and Google Insights | Skin cancer;  Sun protection | Creative communication strategy that uses entertainment-education and social media marketing to engage youth in skin cancer prevention |
| Stapleton et al. (2016) [23] | United States | Facebook  Pinterest  Twitter  Instagram | Not specified | 463 non-Hispanic white participants | Women aged 18–25 years | Cross-sectional study;  Survey | Skin cancer;  Indoor tanning;  Tanning bed | Examining the association between use of social media sites and indoor tanning  behavior |
| Stapleton et al. (2018) [24] | United States | Facebook | Posts | 17 young women | 18 to 25 year-old women | Formative study;  Baseline survey and follow-up survey | Skin cancer;  Indoor tanning beds;  Body acceptance | Healthy body image intervention (behavioral intervention) via Facebook targeting users of indoor tanning beds to reduce their risk of skin cancer – assessment of engagement and acceptability |
| Vraga et al. (2022) [25] | United States | Facebook | Videos | 2173 recruited participants (1348 eligible participants) | adults | Randomized controlled trial;  Questionnaire | Skin cancer; Sunscreen | Assessment of the effects of a news literacy video and real-time corrections to video misinformation related to sunscreen and skin cancer |
| Willoughby et al. (2018) [26] | United States | YouTube,  SnapChat, Instagram, Twitter, Pinterest, Facebook | Not specified (visual-oriented social media platforms) | 502 | college-aged women; from 18 to 29 aged | Online survey | Tanning behaviors (indoor and outdoor tanning) | The role of specific media types in potentially promoting indoor or outdoor tanning |
| Yousaf et al. (2020) [27] | United States | YouTube  Instagram  Pinterest  Facebook  Twitter  Snapchat  Tumblr | Not specified | 130 | Patients of different ages | Cross-sectional study;  survey | Acne | Assessing the impact of social media use on acne treatment |

References:

14. Basch CH, Hillyer GC, Romero RA, MacLean SA, Ethan D. College students’ attitudes and behaviors related to sun safety and appearance in relation to health information-seeking behavior and social media use: cross-sectional study. JMIR Dermatol. Dec 19, 2018;1(2):e10984. [[doi: 10.2196/10984](https://derma.jmir.org/2018/2/e10984/)]

15. Buller DB, Pagoto S, Baker K, Walkosz BJ, Hillhouse J, Henry KL, et al. Results of a social media campaign to prevent indoor tanning by teens: a randomized controlled trial. Prev Med Rep. Jun 2021;22:101382. [doi: 10.1016/j.pmedr.2021.101382] [Medline: 33996394]

16. Buller DB, Pagoto S, Henry KL, Baker K, Walkosz BJ, Hillhouse J, et al. Persisting effects of a social media campaign to prevent indoor tanning: a randomized trial. Cancer Epidemiol Biomarkers Prev. Apr 01, 2022;31(4):885-892. [doi: 10.1158/1055-9965.EPI-21-0059] [Medline: 35064063]

17. Buller DB, Pagoto S, Henry KL, Baker K, Walkosz BJ, Hillhouse J, et al. Effects of engagement with a social media campaign for mothers to prevent indoor tanning by teens in a randomized trial. J Health Commun. Jun 03, 2022;27(6):394-406. [doi: 10.1080/10810730.2022.2113839] [Medline: 35993376]

18. Coups EJ, Manne SL, Pagoto SL, Criswell KR, Goydos JS. Facebook intervention for young-onset melanoma patients and their family members: pilot and feasibility study. JMIR Dermatol. Nov 01, 2018;1(2):e3. [doi: [10.2196/derma.9734](https://derma.jmir.org/2018/2/e3/)]

19. Martel J, Powell E, Murina A. The effect of Instagram and photograph editing on seeking dermatologic care. J Cosmet Dermatol. Oct 16, 2020;19(10):2732-2735. [doi: [10.1111/jocd.13456](https://onlinelibrary.wiley.com/doi/10.1111/jocd.13456)] [Medline: 32333461]

20. Morrison L, Chen C, Torres JS, Wehner M, Junn A, Linos E. Facebook advertising for cancer prevention: a pilot study. Br J Dermatol. Oct 04, 2019;181(4):858-859. [doi: 10.1111/bjd.17993] [Medline: 30972743]

21. Myrick JG, Waldron KA, Cohen O, DiRusso C, Shao R, Cho E, et al. The effects of embedded skin cancer interventions on sun-safety attitudes and attention paid to tan women on Instagram. Front Psychol. Apr 8, 2022;13:838297. [doi: 10.3389/fpsyg.2022.838297] [Medline: 35465513]

22. Pagoto SL, Waring ME, Groshon LC, Rosen AO, Schroeder MW, Goetz JM. Proof-of-concept feasibility trial of a dissonance-based sun safety intervention for young adult tanners. Ann Behav Med. Aug 02, 2022;56(8):830-841. [doi: 10.1093/abm/kaab116] [Medline: 35179176]

23. Stapleton JL, Hillhouse J, Coups EJ, Pagoto S. Social media use and indoor tanning among a national sample of young adult nonHispanic White women: a cross-sectional study. J Am Acad Dermatol. Jul 2016;75(1):218-220. [doi: 10.1016/j.jaad.2016.01.043] [Medline: 27317521]

24. Stapleton JL, Manne SL, Day AK, Levonyan-Radloff K, Pagoto SL. Healthy body image intervention delivered to young women via Facebook groups: formative study of engagement and acceptability. JMIR Res Protoc. Feb 20, 2018;7(2):e54. [doi: 10.2196/resprot.9429] [Medline: 29463495]

25. Vraga EK, Bode L, Tully M. The effects of a news literacy video and real-time corrections to video misinformation related to sunscreen and skin cancer. Health Commun. Nov 12, 2022;37(13):1622-1630. [doi: [10.1080/10410236.2021.1910165](https://www.tandfonline.com/doi/full/10.1080/10410236.2021.1910165)] [Medline: 33840310]

26. Willoughby JF, Myrick JG. Entertainment, social media use and young women’s tanning behaviours. Health Educ J. Dec 23, 2018;78(3):352-365. [doi: [10.1177/0017896918819643](https://journals.sagepub.com/doi/10.1177/0017896918819643)]

27. Yousaf A, Hagen R, Delaney E, Davis S, Zinn Z. The influence of social media on acne treatment: a cross-sectional survey. Pediatr Dermatol. Mar 2020;37(2):301-304. [doi: 10.1111/pde.14091] [Medline: 31944359]

28. Agha-Mir-Salim L, Bhattacharyya A, Hart D, Lewandowska M, Spyropoulou E, Stinson L, et al. A randomised controlled trial evaluating the effectiveness of Facebook compared to leaflets in raising awareness of melanoma and harmful sun-related behaviour among young adults. Eur J Cancer Prev. Jan 2020;29(1):89-91. [doi: [10.1097/CEJ.0000000000000519](https://journals.lww.com/eurjcancerprev/abstract/2020/01000/a_randomised_controlled_trial_evaluating_the.12.aspx)] [Medline: 30998526]

29. Gough A, Hunter RF, Ajao O, Jurek A, McKeown G, Hong J, et al. Tweet for behavior change: using social media for the dissemination of public health messages. JMIR Public Health Surveill. Mar 23, 2017;3(1):e14. [doi: 10.2196/publichealth.6313] [Medline: 28336503]

30. Guckian J, Jobling K, Oliphant T, Weatherhead S, Blasdale K. 'I saw it on Facebook!' Assessing the influence of social media on patient presentation to a melanoma screening clinic. Clin Exp Dermatol. Apr 02, 2020;45(3):295-301. [doi: [10.1111/ced.14100](https://academic.oup.com/ced/article-abstract/45/3/295/6598067?redirectedFrom=fulltext)] [Medline: 31541480]

31. Mingoia J, Hutchinson AD, Gleaves DH, Corsini N, Wilson C. Use of social networking sites and associations with skin tone dissatisfaction, sun exposure, and sun protection in a sample of Australian adolescents. Psychol Health. Dec 2017;32(12):1502-1517. [doi: [10.1080/08870446.2017.1347788](https://www.tandfonline.com/doi/full/10.1080/08870446.2017.1347788)] [Medline: 28691513]

32. Mingoia J, Hutchinson AD, Gleaves DH, Wilson C. The impact of a social media literacy intervention on positive attitudes to tanning: a pilot study. Comput Human Behav. Jan 2019;90:188-195. [doi: [10.1016/j.chb.2018.09.004](https://www.sciencedirect.com/science/article/abs/pii/S0747563218304424?via%3Dihub)]

33. Potente S, McIver J, Anderson C, Coppa K. “It's a beautiful day for cancer”: an innovative communication strategy to engage youth in skin cancer prevention. Soc Mark Q. Sep 01, 2011;17(3):86-105. [doi: [10.1080/15245004.2011.595604](https://journals.sagepub.com/doi/10.1080/15245004.2011.595604)]

34. Damude S, Hoekstra-Weebers JE, van Leeuwen BL, Hoekstra HJ. Melanoma patients' disease-specific knowledge, information preference, and appreciation of educational YouTube videos for self-inspection. Eur J Surg Oncol. Aug 2017;43(8):1528-1535. [doi: [10.1016/j.ejso.2017.06.008](https://www.ejso.com/article/S0748-7983(17)30548-6/abstract)] [Medline: 28684059]

35. Køster B, Thorgaard C, Philip A, Clemmensen I. Sunbed use and campaign initiatives in the Danish population, 2007-2009: a cross-sectional study. J Eur Acad Dermatol Venereol. Nov 09, 2011;25(11):1351-1355. [doi: [10.1111/j.1468-3083.2010.03960.x](https://onlinelibrary.wiley.com/doi/10.1111/j.1468-3083.2010.03960.x)] [Medline: 21711466]

36. Bahaj RK, Alsaggaf ZH, Abduljabbar MH, Hariri JO. The influence of social media on the treatment of acne in Saudi Arabia. Cureus. Mar 2022;14(3):e23169. [doi: 10.7759/cureus.23169] [Medline: 35444887]
