# Supplementary material for: Social Media Potential and Impact on Changing Behaviors and Actions in Skin Health Promotion: Systematic Review
Source: J Med Internet Res. 2025 Jan 6;27:e54241. doi: 10.2196/54241 (PMC11747541; doi:10.2196/54241)
Supplement: Multimedia Appendix 2 [file jmir_v27i1e54241_app2.docx]

Table 3. Findings of included studies.

| Author (year) | Intervention | Assessment of the impact of social media | The impact of public health campaigns | Skin self-examination | Use of cosmetic preparations | Statistical analysis performed | Main finding(s) | Conclusion/Opinion of the author(s) of the study regarding the effectiveness of the strategy for providing information on social media | Quality appraisal |
| --- | --- | --- | --- | --- | --- | --- | --- | --- | --- |
| Agha-Mir-Salim et al. (2020) [28] | + | + | + | - | + | + | - After the SunSafe campaign, there was a statistically significant increase in the level of knowledge (Facebook = 1.82, leaflets = 3.04, P < 0.001);  - The improvement in the knowledge score from leaflets was statistically significantly greater than from Facebook (95% confidence interval: 0.35-2.09, P = 0.0059);  - Participants with lighter skin color had higher levels of knowledge about skin cancer and sun exposure at baseline (P = 0.005, P < 0.05) | Printed leaflets have a greater impact on raising awareness than online platforms such as Facebook among young adults in the UK, so traditional media should not be neglected in health promotion. | high |
| Bahaj et al. (2022) [36] | - | + | - | - | + | + | - The majority of social media users were between 18 and 25 years old and chose social media as their first approach for acne advice;  - The most frequent social media recommendation chosen was to increase water intake;  - There was a significant association between the use  of social media for advice and gender (p-value< 0.001), education level (p-value = 0.002), and severity of  acne (p-value< 0.001) | Social media has had a significant impact on acne treatment, however, much of the advice recommended is not consistent with current guidelines | high |
| Basch et al. (2018) [14] | - | + | - | - | + | + | - Females (odds ratio [OR] 0.42, 95% CI 0.22-0.81) and believers that sun tan improves appearance (OR 0.25, 95% CI 0.10-0.66) were less likely to have “high” sun protection behaviors | Incorporating personal stories and graphic images into skin cancer prevention messages can improve message retention, but knowledge alone does not necessarily influence behavior, so effective interventions focused on skin cancer prevention and sun protection behavior must be multifaceted | high |
| Buller et al. (2021) [15] | + | + | + | - | - | + | - At 12-month follow-up, intervention-group mothers were less permissive about indoor tanning by daughters (unadjusted means = 1.70 [95% CI: 1.59, 1.80] v. 1.85 [1.73, 1.97] [5-point Likert scale], b = -0.152) than control-group mothers;  - At 12-month follow-up, intervention-group mothers reported more communication about avoiding indoor tanning with daughters (4.09 [3.84, 4.35] v. 3.42 [3.16, 3.68] [sum of 7 yes/no items], b = 0.213) than control-group mothers | A social media campaign may be an effective strategy to convince mothers to withhold permission for indoor tanning | high |
| Buller et al. (2022) [16] | + | + | + | - | - | + | - 76.4% of posts received a reaction and/or comment;  - Mothers who engaged with indoor tanning posts were less permissive of daughters' indoor tanning immediately at the conclusion of the campaign (permit indoor tanning: -0.39, p<0.05; facilitate indoor tanning: -0.29, p<0.05) and 6 months after intervention (permit indoor tanning: -0.32, p<0.05; facilitate indoor tanning: -0.31, p<0.05) than mothers who did not engage with posts | Engagement with posts is essential to the success of a social media campaign for preventing indoor tanning | high |
| Buller et al. (2022) [17] | + | + | + | - | - | + | - Intervention-group mothers were less permissive of indoor tanning by daughters (unstandardized coefficient=−0.17, 95% confidence interval [CI], −0.31, −0.03) than control-group mothers;  - Intervention-group mothers had greater self-efficacy to refuse daughter’s indoor tanning requests (0.17, 95% CI, 0.06, 0.29) and lower indoor tanning intentions themselves (−0.18, 95% CI, −0.35, −0.01) than control-group mothers;  - Intervention-group mothers were more supportive of bans on indoor tanning by minors (0.23, 95% CI, 0.02, 0.43) than control-group mothers;  - Intervention-group daughters expressed less positive indoor tanning attitudes than controls (−.16, 95% CI, 0.31, −0.01) | A social media campaign led to a lasting reduction in mothers' leniency towards their daughters' indoor tanning | high |
| Coups et al. (2018) [18] | + | + | - | + | + | + | - Among the 5 consecutive groups conducted, engagement, as measured by comments and likes in response to postings about skin cancer, skin cancer risk factors, total cutaneous exam (TCE), skin self-exam (SSE), and sun protection, increased across the groups;  - Participants positively evaluated the intervention content and approach;  - Preliminary analyses indicated increases in TCE, SSE, and sun protection intentions | A Facebook intervention is a potentially feasible and effective method for increasing sun protection and skin cancer surveillance among people at increased risk for melanoma | high |
| Damude et al. (2017) [34] | + | + | - | + | - | + | - Orally delivered information regarding warning signs, severity, treatment possibilities, and importance of self-inspection was clearest for patients, compared to information in the melanoma brochure;  - According to 77% of patients, YouTube videos regarding self-inspection of the skin and regional lymph nodes had additional value | YouTube videos are an easily accessible and complementary method of patient education | low |
| Gough et al. (2017) [29] | + | + | + | - | - | - | - Greater awareness that skin cancer is the most common form of cancer (pre intervention: 28.4% [95/335] vs. post intervention: 39.3% [168/428] answered "True");  - Melanoma is most serious skin cancer (49.1% [165/336] vs 55.5% [238/429]);  - Improved attitudes toward ultraviolet (UV) exposure and skin cancer with a reduction in agreement that respondents "like to tan" (60.5% [202/334] vs. 55.6% [238/428]) | Social media are an inexpensive, effective method for delivering public health messages | low |
| Guckian et al. (2020) [30] | - | + | + | + | - | + | - Self-examination of lesions was the most common motivator for attending a melanoma screening clinic;  - 1 person described social media as a motivating factor;  - 30 people saw posts from health authorities about skin cancer | Current information delivery strategies are not of the required quality to reach target populations | high |
| Køster et al. (2011) [35] | + | + | + | - | - | + | - The odds ratio (OR) for being a sunbed user in 2009 when compared with 2007 was 0.61 (0.54-0.69);  - in the age group of 15-19 years, the OR was 0.42 (0.30-0.69);  - In 2009 23% of Danes (33% of 15-19-year-olds) reported sunbed use within the past 12 months;  - In 2009 of Danes more than 50% had experienced sunburn caused by a sunbed | The use of tanning beds decreased in parallel with the anti sunbed campaign on social media (the biggest change occurred in the 15-19 age group) | high |
| Martel et al. (2020) [19] | - | + | - | + | - | + | - 145/228 (63.6%) edited a skin lesion, 104/228 (45.6%) whitened teeth, 70/228 (30.7%) altered the size of their body;  - Of those who edited skin lesions, 128/145 (88.3%) edited acne/acne scars;  - Those who felt the editing made them more aware of their skin lesions were significantly more likely to feel they needed to see a dermatologist (P=0.02, 95% CI) | Editing photos of skin lesions/acne scars on Instagram may be a motivating factor to seek dermatological care | high |
| Mingoia et al. (2017) [31] | - | + | - | - | + | + | - Behavior related to tanning (viewing pictures, posting pictures, and liking or sharing content) were significantly associated with more skin tone dissatisfaction, more sun exposure and less sun protection | Posting photos, posting text, viewing photos, viewing text, and liking or sharing tan-related posts on social media are associated with skin cancer risk factors | high |
| Mingoia et al. (2019) [32] | + | + | - | - | - | + | - Significantly decreased positive tanning attitudes in the intervention group compared to the control group;  - Improved media literacy skills in both groups;  - Intervention group participants reported significantly less internalization of a tanned ideal, appearance comparisons, tanning intentions than control group participants | The social media literacy intervention is a strategy to reduce the harmful impact of social media messages about tanning, which to skin cancer prevention | high |
| Morrison et al. (2019) [20] | + | + | + | - | - | + | - The videos received 1288  comments, 11415 reactions and 4201 shares;  - The parody song had the highest engagement  (1050 comments, 7725 reactions, 3875 shares);  - The fact-based video had the lowest engagement  (22 comments, 121 reactions, 70 shares);  - A greater proportion of the intervention group responded “yes” to recalling seeing a  skin cancer prevention video (82/428, 19.2%), compared to the control group (31/449, 6.9%) – difference was statistically significant (N=877, OR=3.19, 95%CI 2.06–4.93, p<0.0001) | Social media advertising can reach target populations and provides an opportunity for targeted public health interventions | high |
| Myrick et al. (2022) [21] | + | + | - | - | + | + | - Women who first viewed the appearance benefits intervention story spent less time visually fixated on Instagram images of tan women than did those who viewed the self-control emotions intervention or control message (p = 0.005, η2p = 0.087);  - Interactions between the intervention conditions and feelings of anticipated pride on both visual attention and sun-safety attitudes | Depending on the emotional responses at Instagram generates, it may promote sun safety attitudes | high |
| Pagoto et al. (2022) [22] | + | + | + | - | + | + | - Retention was 100%;  - Most Healthy Skin (88%) and Healthy Lifestyle participants (91%) created ≥1 post;  - At 4 weeks, Healthy Skin participants reported greater declines in motivation to tan indoors (p =0.0017) and outdoors (p =0.0003);  - At 4 weeks, Healthy Skin participants reported greater increases in motivation to wear sunscreen (p =0.0009) and protective clothing (p =0.0342);  - At 4 weeks, Healthy Skin participants reported greater declines in intentions to tan outdoors in the next year (p =0.0286) | A dissonance-based, social media sun safety intervention is feasible and acceptable | high |
| Potente et al. (2011) [33] | + | + | + | - | + | + | - 25% of people who watched the video passed it on to their friends;  - 44% said the film changed their feelings about sun protection;  - 19% of spontaneous online comments reported an intention to change sun protection behavior | Entertaining, peer-to-peer messages can be used to engage youth with an important health message for skin cancer prevention | high |
| Stapleton et al. (2016) [23] | - | + | - | - | - | + | - Frequency of use of Facebook or Pinterest was not associated with past 12-month indoor tanning;  - Higher rates of indoor tanning were associated with use of Twitter and Instagram among a national sample of young adult women | Twitter and Instagram can be a valuable way to spread skin cancer prevention information to social media users using indoor tanning | high |
| Stapleton et al. (2018) [24] | + | + | - | - | - | + | - The study had a high retention rate (94%, [16/17]);  - Average rates of past 1-month indoor tanning reported following the intervention were lower than the baseline rate (P=0.08, Cohen d=0.47) | An intervention approach using dissonance-inducing content shows potential value for indoor tanning, body image, and skin cancer prevention | high |
| Vraga et al. (2022) [25] | + | + | - | - | + | + | - Video misinformation heightened beliefs in sunscreen myths and reduced acceptance of sunscreen facts and intentions to wear sunscreen compared to a promotional video;  - Real-time user corrections were partially successful in reducing the effects of the misinformation video on beliefs but not intentions | Exposure to a news literacy video did not inoculate people to the misinformation. The ability to correct video misinformation on health issues is important. | high |
| Willoughby et al. (2018) [26] | - | + | - | - | - | + | - Greater magazine use predicted increased indoor tanning;  - Social media use predicted outdoor sunbathing frequency;  - Greater use of social media (Instagram, SnapChat and Pinterest) predicted increased sunbathing | Health communicators should consider specific channels for prevention efforts, particularly social media for targeting sunbathers | high |
| Yousaf et al. (2020) [27] | - | + | - | - | + | + | - 45% consulted social media for acne treatment advice;  - Social media users often tried an OTC (Over the Counter) treatment (81%) or dietary modification (40%);  - 31% of participants consulting social media made changes fully aligned with AAD (American Academy of Dermatology) clinical guidelines | Social media influences acne treatment. Social media users use social media for acne treatment advice. Dermatologists should respond to misinformation | high |

(+) – measures used by the authors

(-) – measures not used by the author

References:

14. Basch CH, Hillyer GC, Romero RA, MacLean SA, Ethan D. College students’ attitudes and behaviors related to sun safety and appearance in relation to health information-seeking behavior and social media use: cross-sectional study. JMIR Dermatol. Dec 19, 2018;1(2):e10984. [[doi: 10.2196/10984](https://derma.jmir.org/2018/2/e10984/)]

15. Buller DB, Pagoto S, Baker K, Walkosz BJ, Hillhouse J, Henry KL, et al. Results of a social media campaign to prevent indoor tanning by teens: a randomized controlled trial. Prev Med Rep. Jun 2021;22:101382. [doi: 10.1016/j.pmedr.2021.101382] [Medline: 33996394]

16. Buller DB, Pagoto S, Henry KL, Baker K, Walkosz BJ, Hillhouse J, et al. Persisting effects of a social media campaign to prevent indoor tanning: a randomized trial. Cancer Epidemiol Biomarkers Prev. Apr 01, 2022;31(4):885-892. [doi: 10.1158/1055-9965.EPI-21-0059] [Medline: 35064063]

17. Buller DB, Pagoto S, Henry KL, Baker K, Walkosz BJ, Hillhouse J, et al. Effects of engagement with a social media campaign for mothers to prevent indoor tanning by teens in a randomized trial. J Health Commun. Jun 03, 2022;27(6):394-406. [doi: 10.1080/10810730.2022.2113839] [Medline: 35993376]

18. Coups EJ, Manne SL, Pagoto SL, Criswell KR, Goydos JS. Facebook intervention for young-onset melanoma patients and their family members: pilot and feasibility study. JMIR Dermatol. Nov 01, 2018;1(2):e3. [doi: [10.2196/derma.9734](https://derma.jmir.org/2018/2/e3/)]

19. Martel J, Powell E, Murina A. The effect of Instagram and photograph editing on seeking dermatologic care. J Cosmet Dermatol. Oct 16, 2020;19(10):2732-2735. [doi: [10.1111/jocd.13456](https://onlinelibrary.wiley.com/doi/10.1111/jocd.13456)] [Medline: 32333461]

20. Morrison L, Chen C, Torres JS, Wehner M, Junn A, Linos E. Facebook advertising for cancer prevention: a pilot study. Br J Dermatol. Oct 04, 2019;181(4):858-859. [doi: 10.1111/bjd.17993] [Medline: 30972743]

21. Myrick JG, Waldron KA, Cohen O, DiRusso C, Shao R, Cho E, et al. The effects of embedded skin cancer interventions on sun-safety attitudes and attention paid to tan women on Instagram. Front Psychol. Apr 8, 2022;13:838297. [doi: 10.3389/fpsyg.2022.838297] [Medline: 35465513]

22. Pagoto SL, Waring ME, Groshon LC, Rosen AO, Schroeder MW, Goetz JM. Proof-of-concept feasibility trial of a dissonance-based sun safety intervention for young adult tanners. Ann Behav Med. Aug 02, 2022;56(8):830-841. [doi: 10.1093/abm/kaab116] [Medline: 35179176]

23. Stapleton JL, Hillhouse J, Coups EJ, Pagoto S. Social media use and indoor tanning among a national sample of young adult nonHispanic White women: a cross-sectional study. J Am Acad Dermatol. Jul 2016;75(1):218-220.[doi: 10.1016/j.jaad.2016.01.043] [Medline: 27317521]

24. Stapleton JL, Manne SL, Day AK, Levonyan-Radloff K, Pagoto SL. Healthy body image intervention delivered to young women via Facebook groups: formative study of engagement and acceptability. JMIR Res Protoc. Feb 20, 2018;7(2):e54. [doi: 10.2196/resprot.9429] [Medline: 29463495]

25. Vraga EK, Bode L, Tully M. The effects of a news literacy video and real-time corrections to video misinformation related to sunscreen and skin cancer. Health Commun. Nov 12, 2022;37(13):1622-1630. [doi: [10.1080/10410236.2021.1910165](https://www.tandfonline.com/doi/full/10.1080/10410236.2021.1910165)] [Medline: 33840310]

26. Willoughby JF, Myrick JG. Entertainment, social media use and young women’s tanning behaviours. Health Educ J. Dec 23, 2018;78(3):352-365. [doi: [10.1177/0017896918819643](https://journals.sagepub.com/doi/10.1177/0017896918819643)]

27. Yousaf A, Hagen R, Delaney E, Davis S, Zinn Z. The influence of social media on acne treatment: a cross-sectional survey. Pediatr Dermatol. Mar 2020;37(2):301-304. [doi: 10.1111/pde.14091] [Medline: 31944359]

28. Agha-Mir-Salim L, Bhattacharyya A, Hart D, Lewandowska M, Spyropoulou E, Stinson L, et al. A randomised controlled trial evaluating the effectiveness of Facebook compared to leaflets in raising awareness of melanoma and harmful sun-related behaviour among young adults. Eur J Cancer Prev. Jan 2020;29(1):89-91. [doi: [10.1097/CEJ.0000000000000519](https://journals.lww.com/eurjcancerprev/abstract/2020/01000/a_randomised_controlled_trial_evaluating_the.12.aspx)] [Medline: 30998526]

29. Gough A, Hunter RF, Ajao O, Jurek A, McKeown G, Hong J, et al. Tweet for behavior change: using social media for the dissemination of public health messages. JMIR Public Health Surveill. Mar 23, 2017;3(1):e14. [doi: 10.2196/publichealth.6313] [Medline: 28336503]

30. Guckian J, Jobling K, Oliphant T, Weatherhead S, Blasdale K. 'I saw it on Facebook!' Assessing the influence of social media on patient presentation to a melanoma screening clinic. Clin Exp Dermatol. Apr 02, 2020;45(3):295-301. [doi: [10.1111/ced.14100](https://academic.oup.com/ced/article-abstract/45/3/295/6598067?redirectedFrom=fulltext)] [Medline: 31541480]

31. Mingoia J, Hutchinson AD, Gleaves DH, Corsini N, Wilson C. Use of social networking sites and associations with skin tone dissatisfaction, sun exposure, and sun protection in a sample of Australian adolescents. Psychol Health. Dec 2017;32(12):1502-1517. [doi: [10.1080/08870446.2017.1347788](https://www.tandfonline.com/doi/full/10.1080/08870446.2017.1347788)] [Medline: 28691513]

32. Mingoia J, Hutchinson AD, Gleaves DH, Wilson C. The impact of a social media literacy intervention on positive attitudes to tanning: a pilot study. Comput Human Behav. Jan 2019;90:188-195. [doi: [10.1016/j.chb.2018.09.004](https://www.sciencedirect.com/science/article/abs/pii/S0747563218304424?via%3Dihub)]

33. Potente S, McIver J, Anderson C, Coppa K. “It's a beautiful day for cancer”: an innovative communication strategy to engage youth in skin cancer prevention. Soc Mark Q. Sep 01, 2011;17(3):86-105. [doi: [10.1080/15245004.2011.595604](https://journals.sagepub.com/doi/10.1080/15245004.2011.595604)]

34. Damude S, Hoekstra-Weebers JE, van Leeuwen BL, Hoekstra HJ. Melanoma patients' disease-specific knowledge, information preference, and appreciation of educational YouTube videos for self-inspection. Eur J Surg Oncol. Aug 2017;43(8):1528-1535. [doi: [10.1016/j.ejso.2017.06.008](https://www.ejso.com/article/S0748-7983(17)30548-6/abstract)] [Medline: 28684059]

35. Køster B, Thorgaard C, Philip A, Clemmensen I. Sunbed use and campaign initiatives in the Danish population, 2007-2009: a cross-sectional study. J Eur Acad Dermatol Venereol. Nov 09, 2011;25(11):1351-1355. [doi: [10.1111/j.1468-3083.2010.03960.x](https://onlinelibrary.wiley.com/doi/10.1111/j.1468-3083.2010.03960.x)] [Medline: 21711466]

36. Bahaj RK, Alsaggaf ZH, Abduljabbar MH, Hariri JO. The influence of social media on the treatment of acne in Saudi Arabia. Cureus. Mar 2022;14(3):e23169. [doi: 10.7759/cureus.23169] [Medline: 35444887]
